# Comprehensive nursing practice in Primary Health Care

**DOI:** 10.4102/phcfm.v2i1.189

**Published:** 2010-08-16

**Authors:** Wilma Kotzé

**Affiliations:** 1 Department of Nursing Science, Nelson Mandela Metropolitan University, Port Elizabeth, South Africa

Urbanisation and the mal-distribution of health care professionals in South Africa over decades have led to nurses functioning beyond the legal parameters of their professional scope of practice. Forced by circumstances, nurses and midwives, in the interest of patients, and because of the non-availability of the relevant practitioners, often had no other choice but to accept and perform health care duties traditionally belonging to doctors, pharmacists and selected supplementary health care professionals. The Nursing Act had to be amended in the 1980s to make provision for registered professional nurses, in certain circumstances, to carry out functions normally performed by medical practitioners or pharmacists: Section 38A was inserted in the Nursing Act, No. 50 of 1978, through promulgation of the Amendment Act, No. 71 of 1981. This amendment became a reality through consultation between the nursing, medical and pharmacy professions, in cooperation with the Department of Health at the time.

The nature of this manual reflects not only the above *spirit of concern that transcends defined professional boundaries* amongst the key role players in health care provision, but also understanding of *empowerment as an essential key to transform the preparation of clinical nurse practitioners*, within the realities of the health care needs of the South African population today. The understanding and recognition of the potential inherent in the nursing role in health care is refreshing and needs to be applauded.

In this comprehensive manual, prepared by an impressive number of authors from a number of health professions and wide variety of fields of practice – health service and academic – the focus is on development of clinical skills. Sections concisely addressing the management of different processes of practice in primary health care scenarios and considerations such as, *inter alia*, research and teaching and learning, are also included, adding to the comprehensive nature of the manual. The style of writing and the presentation of content are reader- and learner-friendly. As a whole it is a well-prepared, neatly presented publication that offers enjoyable hours of perusing.

In remote and informal settings without the traditional structure, equipment and support systems of practice in institutional health care, nurses could be confronted with challenges demanding decision-making and skills which they often were ill-prepared for. To the newcomer, especially the newly qualified beginning practitioner, who lacks experience of the realities of practice and the know-how to manage the dynamics of primary health care practice, this presents a daunting experience that could lead to tremendous emotional trauma and disillusionment. The manual, with its comprehensive content and design, presents an excellent compass to nursing practitioners of all levels on their journey of learning to understand and cope with such realities and challenges.

The aim of the book is to empower students, qualified nurses and managers and teachers of nursing as well as other health care students and professionals to:

- gain understanding of the practice field of primary health care and its dynamics and challenges
- cope with the clinical and other demands of practice in primary health care
- develop professionally towards confident, assertive practice in the health care team

The *South African Clinical Nurse Practitioner’s Manual* is an excellent addition to health care literature, well worth acquiring and studying. The authors and especially the editors are congratulated on the quality of content and the neatness of presentation.

**Note:** Professor Wilna Kotzé is an Emeritus Professor at the Nelson Mandela Metropolitan University, Port Elizabeth, South Africa. This book review has been previously published by Van Schaik in January 2010.

**FIGURE 1:**
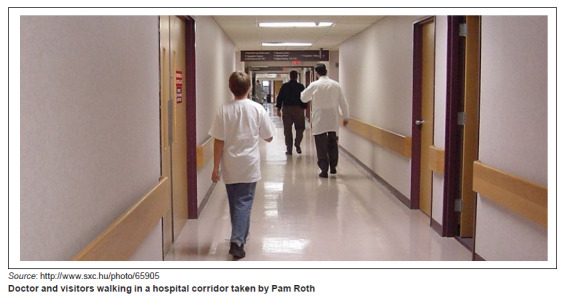
Doctor and visitors walking in a hospital corridor taken by Pam Roth. *Source:* http://www.sxc.hu/photo/65905

